# Spectroscopic Studies on Organic Matter from Triassic Reptile Bones, Upper Silesia, Poland

**DOI:** 10.1371/journal.pone.0151143

**Published:** 2016-03-15

**Authors:** Dawid Surmik, Andrzej Boczarowski, Katarzyna Balin, Mateusz Dulski, Jacek Szade, Barbara Kremer, Roman Pawlicki

**Affiliations:** 1 Faculty of Earth Science, University of Silesia, Będzińska 60, 41–200, Sosnowiec, Poland; 2 Park of Science & Human Evolution, 1 Maja 10, 46–040, Krasiejów, Poland; 3 A. Chełkowski Institute of Physics, University of Silesia, Uniwersytecka 4, 40–007, Katowice, Poland; 4 Silesian Centre for Education and Interdisciplinary Research, 75 Pułku Piechoty 1A, 41–500, Chorzow, Poland; 5 Institute of Material Science, University of Silesia, 75 Pułku Piechoty 1A, 41–500, Chorzow, Poland; 6 Institute of Paleobiology, Polish Academy of Science, Twarda 51/55, 00–818, Warszawa, Poland; 7 Department of Histology, Jagiellonian University Medical College, Kopernika 7, 31–034, Kraków, Poland; Naturhistoriska riksmuseet, SWEDEN

## Abstract

Fossil biomolecules from an endogenous source were previously identified in Cretaceous to Pleistocene fossilized bones, the evidence coming from molecular analyses. These findings, however, were called into question and an alternative hypothesis of the invasion of the bone by bacterial biofilm was proposed. Herewith we report a new finding of morphologically preserved blood-vessel-like structures enclosing organic molecules preserved in iron-oxide-mineralized vessel walls from the cortical region of nothosaurid and tanystropheid (aquatic and terrestrial diapsid reptiles) bones. These findings are from the Early/Middle Triassic boundary (Upper Roetian/Lowermost Muschelkalk) strata of Upper Silesia, Poland. Multiple spectroscopic analyses (FTIR, ToF-SIMS, and XPS) of the extracted "blood vessels" showed the presence of organic compounds, including fragments of various amino acids such as hydroxyproline and hydroxylysine as well as amides, that may suggest the presence of collagen protein residues. Because these amino acids are absent from most proteins other than collagen, we infer that the proteinaceous molecules may originate from endogenous collagen. The preservation of molecular signals of proteins within the "blood vessels" was most likely made possible through the process of early diagenetic iron oxide mineralization. This discovery provides the oldest evidence of *in situ* preservation of complex organic molecules in vertebrate remains in a marine environment.

## Introduction

The conventional wisdom states that no original organic components remains associated with Mesozoic vertebrate bones over geological time. It is based on models using unrealistically harsh chemical conditions as proxies for time [1]. However, half a century ago, one of us (RP) was the first to demonstrate, by describing fossilized cells, collagen fibrils and vessels from Cretaceous dinosaur bones from the Gobi Desert [2], that this conventional wisdom may not hold for all fossils. Beginning in the 1970s, Pawlicki documented histochemical reactions of glycosaminoglycans [3], lipids [4], and nucleic acids [5] in dinosaur bones. Later, various amino acids were extracted from 150 Ma old sauropod bones by Gurley et al. [6]. Muyzer et al. [7] identified remains of osteocalcin, non-collagenous bone matrix protein in dinosaurs, using radioimmunological assays. Recently, Schweitzer and her colleagues, following up on these early investigations, identified soft tissues in dinosaur bones consistent with collagenous matrices, bone cells (osteocytes), blood vessels, and intravascular contents high in iron. The morphological studies were supported by *in situ* immunological assays and MS/MS sequence data that identified proteins consistent with a vertebrate origin [8–15]. Reports of preserved organic compounds in dinosaurs have been criticized due to the possible presence of bacterial biofilms [16] and other forms of contamination as a potential source of organic matter (compare in [17]). However, the recovery of both sequences for, and antibody binding to, histone proteins eliminates a microbial source [12].

Molecular taphonomy, a branch of modern vertebrate paleobiology, addresses alterations of molecules in natural environments over geological time scales. This emergent discipline has been made possible by the advent of highly sensitive and accurate high-resolution analytical methods, including spectroscopy and mass spectrometry. These methods have been employed to support the hypothesis that complex organic molecules can survive, under certain conditions, over long periods of time. However, interpretation of data from a single method is not conclusive. For example, amide signals recovered by synchrotron radiation Fourier transform infrared spectroscopy (SR-FTIR) in fast-growing embryonic bones of Early Jurassic dinosaurs in China [18] may suggest the preservation of bone proteins. However, according to one of the authors, the spectra interpreted as amide may have been misconstrued [K. Stein, personal communication]. Protein remains were detected from the femur of a Cretaceous mosasaur from Belgium using various methods [19]. Time of Flight Secondary Ion Mass Spectrometry (ToF-SIMS) was used successfully to identify amino acid residues [9, 10], [20–22], heme-derived compounds [22, 23] and melanin pigment [24, 25].

This paper summarizes the results of research on molecules which may indicate a proteinaceous source associated with goethite particles extracted from the bones of Early/Middle Triassic reptiles in southern Poland. The Triassic bones had not been studied previously for potential preservation of organic molecules. During such a long deposition the bones were subjected to various diagenetic processes, most of which negatively affected their preservation. However, diagenetic processes not always have to remove organic signals from bones completely. It has been observed that relatively early mineralization can protect delicate organic material from complete degradation [26, 27]. Minerals that nucleate directly on organic material block or limit the accessibility of enzymes involved in degradation. Thus, microcrystalline minerals deposited on an organic template may form a mineral “cast”, stabilizing the molecules and providing added resistance to long-term biological and physical degradation. Other factors, such as bone structure and the thickness and geochemical properties of sediment, play a crucial role in the preservation of organic matter. Therefore, even in the case of very old bones, original organic molecules can be preserved.

## Geological Settings

The bone samples are from fossiliferous beds at the boundary of the uppermost part of the Röt (Myophoria Beds) [28] and the lowermost part of the Lower Muschelkalk, known also as the Limestone with Entolium and Dadocrinus Unit (Lower Gogolin Formation, see also [29]). These bone-bearing limestones occur in Gogolin and Żyglin in Upper Silesia, southern Poland (S1 Fig). The age of the Gogolin Formation (formerly Gogolin Beds), as specified on the basis of lithostratigraphic [30], biostratigraphic [31] and more recent magnetostratigraphic [32, 33] data, has been dated as Early/Middle Triassic, 247.2 Ma (an absolute age, according to the IUGS International Chronostratigraphic Chart v. 2015/01, http://www.stratigraphy.org). Paleomagnetic studies date the Myophoria Beds and the first 5 m of the Limestone with Entolium and Dadocrinus Unit as Olenekian in age [33]. The sediments were deposited on a carbonate platform situated in the southern part of a shallow epicontinental sea [34, 35].

## Material and Methods

The samples of *Nothosaurus* sp. (Sauropterygia) bones (humerus, WNoZ/s/7/166; femur SUT-MG/F/Tvert/15) and the *Protanystropheus* sp. (Archosauromorpha) vertebral centrum (SUT-MG/F/Tvert/2) derive from the fossiliferous beds of the Limestone with Entolium and Dadocrinus Unit, the lowest part of the Gogolin Formation.

The vertebral centrum of *Protanystropheus* (SUT-MG/F/Tvert/2) and the nothosaur femur (SUT-MG/F/Tvert/15), are from the historical collection of the Catholic priest Eduard Kleemann, and are now deposited in the Museum of Geological Deposits, Faculty of Mining and Geology, Silesian University of Technology, Gliwice, Poland. Triassic vertebrate fossils were collected by Father Kleemann at the turn of the 19^th^ and 20^th^ centuries. The specimens SUT-MG/F/Tvert/2 and SUT-MG/F/Tvert/15 are labeled “Gogolin,” indicating the town of Gogolin, near Opole (Opole Voivodeship, Krapkowice County), as the fossils’ locality.

The humerus (WNoZ/s/7/166) is from the Entolium and Dadocrinus Unit in the Żyglin quarry in the town of Miasteczko Śląskie, near Tarnowskie Góry (Silesian Voivodeship). All permission required for collecting fossils in the Żyglin quarry was obtained through an agreement with the Regional Directorate for Environmental Protection in Katowice. The specimen is now deposited in the Museum of Earth Science, Faculty of Earth Science, University of Silesia, Sosnowiec, Poland.

A recent marine iguana femur (GIUS-12-3628), used in our study as a reference sample, is now kept at the Department of Paleontology and Stratigraphy, Faculty of Earth Sciences, University of Silesia. This femur belonged to an individual Galapagos marine iguana that died from natural causes, and was collected as an isolated element with the permission of the appropriate local authorities. All of these specimens are publicly deposited and accessible to others in permanent repositories.

Marine nothosaurs from the Early/Middle Triassic boundary of Southern Poland are usually preserved as isolated bones and represented mainly by medium-size species. Most likely they are represented by *Nothosaurus* cf. *marchicus* (according to personal observations by DS; see also [36]). Terrestrial tanystropheids, represented by *Protanystropheus antiquus*, lived and fed in intertidal zones (compare in [37]). The occurrence of these animals is relatively early in the European Basin (compare in [38–41]; personal observation by DS).

**Fig 1:**
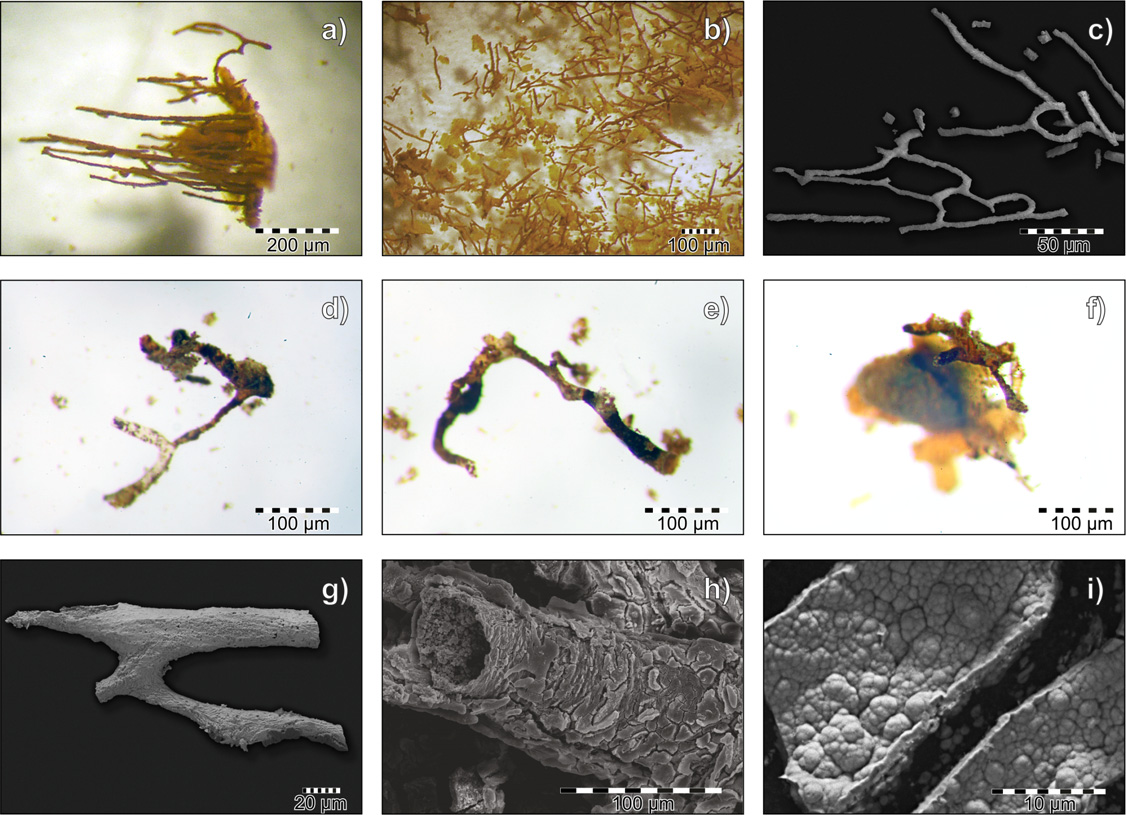
Demineralized blood vessel from fossil samples. Stereoscopic and ESEM microscope images of blood vessels: a) partially demineralized bone sample from the near-cortical region shows parallel-oriented fossilized blood vessels (SUT-MG/F/Tvert/2 sample) in stereoscopic microscope image; b) fossilized “floating” blood vessels from sample SUT-MG/F/Tvert/2 during the demineralization (decalcification) process in EDTA solution in stereoscopic microscope image; c) ESEM image of bifurcated blood vessels mounted on a carbon conductive tab (WNoZ/s/7/166 sample); d-f) isolated branch-like-shaped blood vessels (WNoZ/s/7/166 sample) in stereoscopic microscope images; g) ESEM image of fossilized blood vessel mounted on carbon conductive tab; h) ESEM images of magnified fragment of a mineralized blood vessel with preserved tubular morphology from a demineralized part of bone from specimen WNoZ/s/7/166; i) ESEM image of heavily mineralized, damaged walls of a blood vessel (SUT-MG/F/Tvert/2) with nodular-form goethite crystals, mounted on a carbon conductive tab.

**Fig 2:**
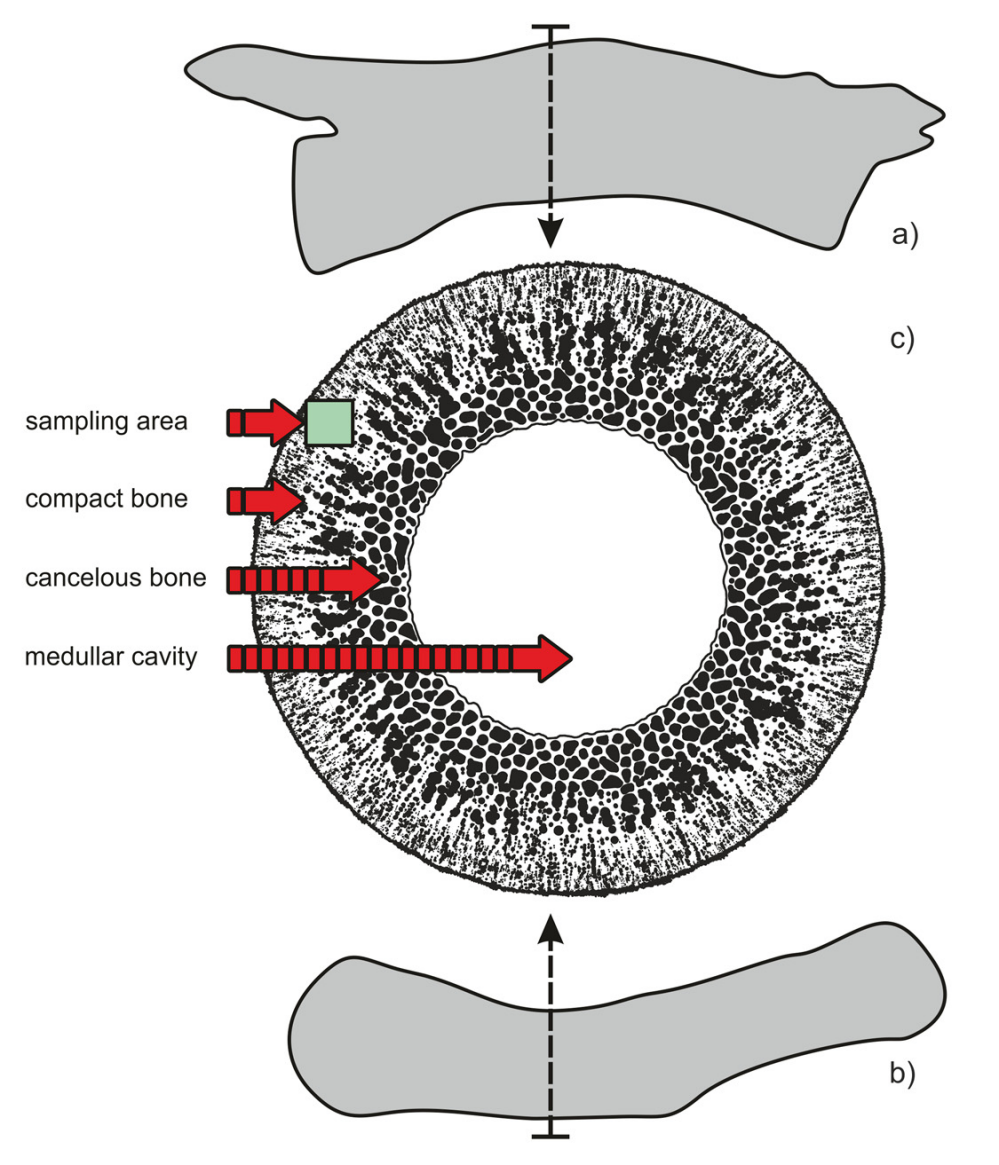
Schematic illustration of sampling area for thin sectioning and demineralization. a) Silhouette of *Protanystropheus* vertebra (SUT-MG/F/Tvert/2 sample) with an indication of the sectioning area; b) silhouette of *Nothosaurus* humerus (WNoZ/s/7/166 sample) with an indication of sampling; c) an idealized schematic cross section of bone with a sampling area within dense, cortical bone tissue.

The bone samples of Triassic reptiles were analyzed in terms of preservation of organic matter residues because preliminary morphological studies (light microscopy and ESEM) revealed the occurrence of vessel-like structures in the cortical part of bone in several samples (Fig 1). The densest areas of cortical (compact) bone of the samples (Fig 2) were chosen for analysis in order to minimalize the risk of microbial contamination from the medullar cavity and from outside. The analyses of “blood vessels” were performed on a partially demineralized (phosphate phase removed) fragment of the *Nothosaurus* humerus and a thin section from a massive cortical part of the *Protanystropheus* cervical vertebra. After these morphological studies, the mineralogical composition of fossil bones was examined using X-ray diffraction (XRD) and subjected to detailed elemental study using an electron dispersive spectrometer (EDS) microanalyzer coupled with ESEM. In the next step, X-ray photoelectron spectroscopy (XPS), infrared spectroscopy (FTIR) and mass spectrometry (ToF-SIMS) techniques were applied to determine types of chemical bonds and to identify iron-mineralized organic matter within fossilized “bones vessels”.

All of the studied specimens possess a documented storage history and have never been glued or treated with any contemporary organic-based materials. The outermost surface contaminants were removed prior to the analyses and the samples were rinsed several times according to the protocols presented herein. All studied fossil bones, as well as control samples, were purified by means of incubation in methanol solution (Methanol:Dichloromethane, CH_4_O:CH_2_Cl_2_, 1:1, Sigma-Aldrich, USA) to remove surface contaminants and rinsed several times with deionized water.

Three types of preparation were performed: **(1)** powdering, **(2)** thin sectioning, **(3)** demineralization.

### Powdering

Fragments of compact bone obtained from the cortical region in the middle shaft of the *Nothosaurus* humerus and *Protanystropheus* vertebral centrum were triturated to an analytical fraction (5‒10μm) in an agate mortar for X-ray powder diffraction and FTIR analysis. A recent control sample of a marine iguana femur was frozen in liquid nitrogen (LN, ‒195.8°C), and then triturated to XRD and FTIR analyses.

### Thin Sectioning

The analyses were performed on covered and uncovered (polished) thin sections about 30 μm thick. Bone fragments were embedded in Araldite 2020 epoxy resin (Huntsman Advanced Materials, USA). The thin sections were polished with silicon carbide (SiC) and aluminum oxide (Al_2_O_3_) papers. Then, the surface was cleaned using diamond paste. Additionally, prior to each analysis, the thin-section surface was cleaned with isopropyl alcohol, 99.9% pure (Sigma-Aldrich, USA), to remove the outermost contaminants.

### Sample Demineralization

A small bone fragment of sample WNoZ/s/7/166 (46.1 mg) was mounted on the top of a vacuum filtration kit (Sartorius AG, Germany) on a sterile Whatman Anodisc 0.02-μm aluminum oxide (Al_2_O_3_) membrane filter (Anopore Inorganic Membranes, GE Healthcare Life Science, USA). The samples were incubated in 0.5M EDTA agent (pH 8.0, filtered by a 0.45-μm Millipore sterile membrane, Merck Millipore, Germany) at room temperature, with two changes of EDTA dilution per day, and rinsed in ultra-pure deionized water with conductivity of 0.05 μS/cm (Elix Essential Water Purification System, Merck Millipore, Germany) several times to remove contaminants. The EDTA dilution and deionized water were removed by manual vacuum pump filtration (PHYWE Systeme GmbH und Co. KG, Germany). We excluded disodium ethylenediaminetetraacetic agent (EDTA) as a source of amino acids by comparing FTIR spectra of pure EDTA salts with sample spectra (S2 Fig).

A residuum containing vessel-like as well as bone-cell-like structures and amorphous reddish-brown mineral material were dried in a vacuum desiccator under sterile conditions at room temperature. Some of the isolated”blood vessels” were manually picked up and separated from the residuum, mounted on carbon conductive tabs to be analyzed in ESEM with and without coating. Another portion of the sample was powdered for XRD and FTIR analyses. The remaining portion of the sample was mounted on a molybdenum holder and placed in a high-vacuum chamber for chemical analysis using ToF-SIMS and XPS.

## Experimental Part

### Optical Microscopy

Optical measurements were carried out using an Olympus BX51 polarizing microscope equipped with an Olympus SC30 camera and a halogen light source, both installed at the Department of Geochemistry, Mineralogy and Petrography, University of Silesia. Optical micrographs were collected using Cell^A 5.1 software (Olympus Soft Imaging Solutions GmbH) using a UMPlanFI 10× objective and an aperture of 0.30.

### Environmental Scanning Electron Microscopy (ESEM)

ESEM images were performed on a Philips XL30 ESEM/EDAX, installed at the Laboratory of Scanning Electron Microscopy, Faculty of Earth Science, University of Silesia, and equipped with an EDAX Sapphire energy-dispersive X-ray spectroscope to analyze the morphology and chemical composition of isolated fossilized “vessels”. The measurements were done on gold-coated bone residuum (high vacuum, accelerated voltage 15 kV) and uncoated thin sections (low vacuum, acc. voltage 15 kV).

### X-ray Diffraction (XRD)

X-ray diffraction analyses (XRD) were undertaken to investigate bone mineral content. We used a PANalytical X'Pert PRO MPD PW 3040/60 diffractometer at the Laboratory of X-ray Diffraction, Faculty of Earth Science, University of Silesia. Quantitative phase content and crystallographic parameters were calculated using the Rietveld Module in HighScore Plus software with the ICDD PDF-4+ pattern database. X-ray diffraction analysis was performed for powdered bone samples as well as for powdered extracted fossilized “blood vessels”. The powder was placed in a reflection-free silicon base in the analyzed area and mounted in a sample changer. The measurements were carried out using the following parameters: source of radiation, Cu K_α1_ (λ = 1.540598 Å); nickel filter, 0.02 mm; voltage, 45 kV; current, 30 mA; scan range, 2.5‒80° 2Θ; step size, 0.01° 2Θ; counting time, 600 s; detector, X'Celerator; analysis time, 6 h.

### Infrared Spectroscopy (FTIR)

Infrared spectroscopy (FTIR) was performed using an Agilent Cary 660 FTIR spectrometer equipped with a standard source and a DTGS Peltier-cooled detector installed at the Department of Biophysics and Molecular Physics, Institute of Physics, University of Silesia. All spectra were accumulated with a spectral resolution of 4 cm^-1^ and recorded by accumulating 16 scans. The baseline correction and fitting analysis by Voigt function for each spectrum were performed using the GRAMS software package. The spectra were collected using a GladiATR diamond accessory (Pike Technologies) in the 4000–400 cm^-1^ range.

### Time of Flight Secondary Ion Mass Spectrometry (ToF-SIMS)

ToF-SIMS experiments were performed using a ToF-SIMS 5 (IONTOF GmbH, Münster, Germany) reflectron-type spectrometer equipped with a bismuth liquid metal ion gun (Bi^+^ and Bi_3_^++^ primary beams) installed at the Department of Solid State Physics, Institute of Physics, University of Silesia. The measurements were performed at room temperature in ultrahigh vacuum conditions (~2‒5∙10^−9^ mbar). High-resolution mass spectra were obtained using a focused high-energy primary ion beam (pulsed 30 keV Bi^+^ or Bi_3_^++^ ions at an ion current of ~1pA and 0.1pA, respectively) aimed at the sample surface at an angle of 45° relative to the surface normal, causing emission of secondary ions. Because structurally different molecules may have almost identical masses, all measurements were performed at high mass resolution mode, given herein an accuracy of 0.01 Da. Positive and negative secondary ion spectra and distribution maps for selected ions were collected by rastering the bismuth ion beam across the regions of interest with an *m/z* range of 1‒800 Da. The size of the areas analyzed varied from 50×50 to 500×500 μm, depending on the region of interest. The mass spectra were internally calibrated using CH_3_^+^, C_2_H_3_^+^, C_2_H_5_^+^, C_3_H_7_^+^, and C_4_H_9_^+^ ions for measurements performed for positive polarity and C^-^, CH^-^, C_2_^-^, C_2_H^-^, C_3_^-^, and C_3_H^-^ ions for negative polarity. Line broadening and thus reduction of mass resolution m/Δm appeared as a result of sample morphology and surface charging. The surfaces of the analyzed samples were neutralized with the use of an electron flood gun. Nevertheless, mass resolution varied depending on polarity or/and sample morphology at the level of 5,000‒9,000. To avoid the impact of contamination, the analyses were performed on surfaces cleaned in the vacuum chamber with the use of a cesium ion gun (Cs at 2 keV and 100 nA rastered over an area typically several times larger than the region of interest; the estimated depth of the removed surface was about 2 μm). Although etching the sample surface with the cesium gun may cause fragmentation of primary molecules present in the specimens, removal of surface contamination was deemed justified. Analysis of the ToF-SIMS spectra, for both negative and positive polarity, enabled the identification of several dozen organic secondary ion species. A number of peaks which, by definition, are organic in nature (showing a mass excess) may be assigned to the secondary ion species typically observed in amino acid mass spectra (see ***Results***). The absence of all molecular peaks in the mass spectrum for a particular amino acid may result from Cs etching, the performance of the ToF-SIMS measurements above the static limit, or significant degradation of the organic molecules in the analyzed specimens. Nevertheless, the presence of amino-acid-related fragment ions was confirmed for both specimens in both polarities, and a number of characteristic peaks showing a mass excess were assignable to the secondary ion species of specific amino acids (see ***Results***). Moreover, taking into account the sample preparation, specifically Cs etching, it was determined that the detected secondary ion originated from the surface of the specimens, not from surface contamination. The analysis of the high mass resolution secondary ion mass spectra, together with the distribution maps of selected secondary ions, enabled the unambiguous determination of ion location in the analyzed area.

**Fig 3:**
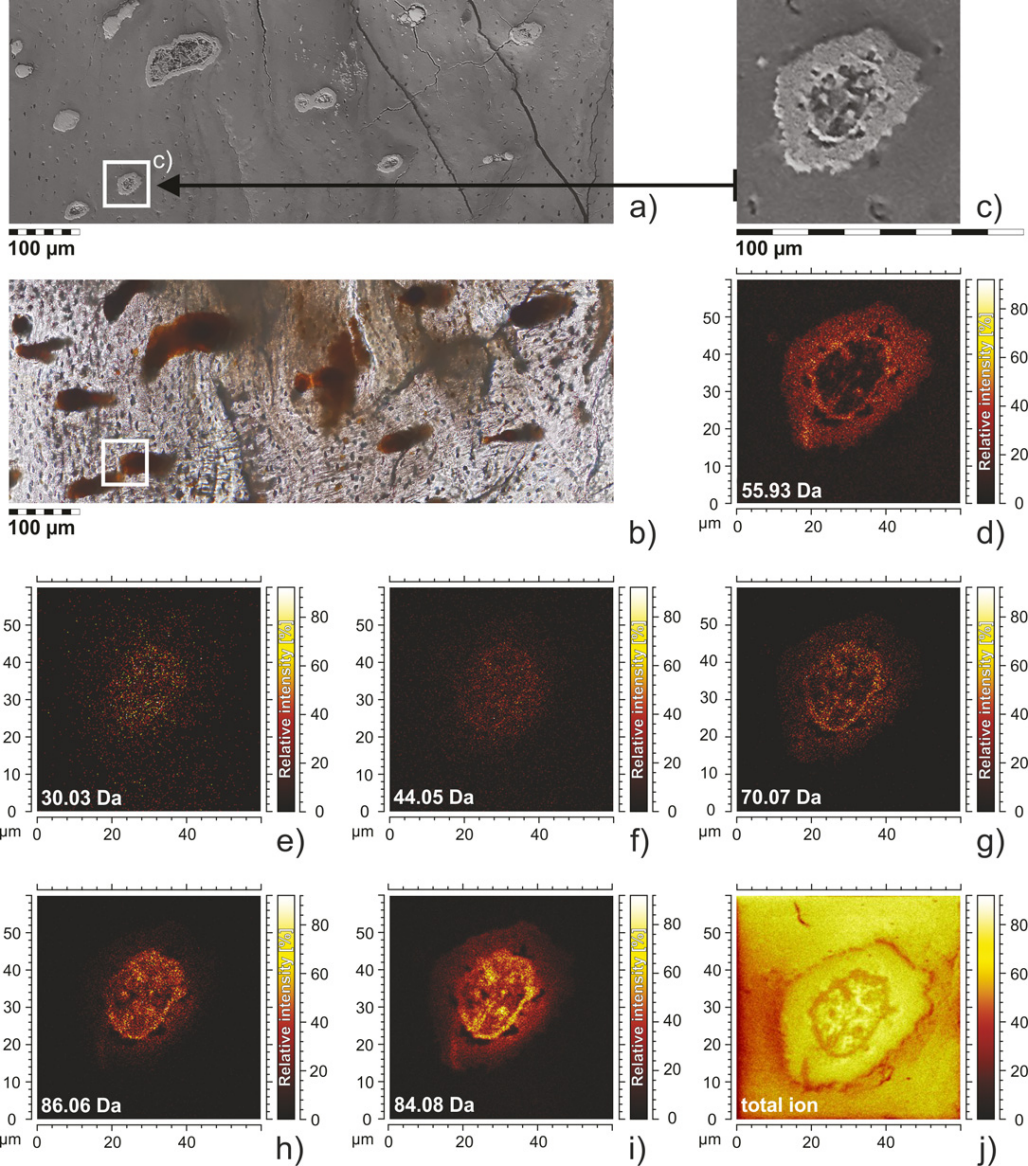
The structure and molecular composition of the fossilized blood vessel sections of SUT-MG/F/Tvert/2. ESEM images and ToF-SIMS fast imaging mode mapping of blood vessels sections displaying their tubular structure. a) ESEM image of thin section showing fossilized blood vessels; analyzed area marked by rectangle. b) the same thin section in optical microscopy; analyzed area marked. c) blood vessel in SEM image, enlarged part of Fig 3a shows location of ToF-SIMS mapping; d‒j) ToF-SIMS ion distribution maps generated for the selected masses corresponding to iron (55.86 Da) and amino acid ions: 30.03 Da–CH_4_N^+^ (glycine or proline), 44.05 Da–C_2_H_6_N^+^ (alanine), 70.07 Da–C_4_H_8_N^+^ (proline), 86.06 Da–C_4_H_8_NO^+^ (hydroxyproline), 84.08 Da–C_5_H_10_N^+^ (lysine) and total ion image (Fig 3j) in positive polarity. The distribution of iron (Fig 3d) within the vessel section overlaps with the distribution of ions.

High lateral resolution maps of ion distribution were obtained by applying the Fast Imaging Mode of the ToF-SIMS spectrometer. In this mode the mass resolution is significantly lowered (m/Δm ~100), which in practice means an inability to determine the presence of particular molecular ion species and/or to distinguish distributions of molecular ions with closely adjacent masses. However, since we performed measurements from the same areas in high mass resolution and high lateral resolution modes we may ascribe particular ions distribution maps to particular ion. We assumed that, since the peak indicating the presence of an ion assigned to a particular amino acid is characterized by the dominant intensity in high mass resolution mode, the related peak obtained from measurements in high lateral resolution mode would also be dominated by the presence of that ion. Hence the distribution maps for particular masses presented in Fig 3 are related to particular amino acid fragments.

### X-Ray Photoelectron Spectroscopy (XPS)

X-ray Photoelectron Spectroscopy (XPS) was performed using an XPS PHI 5700 spectrometer from Physical Electronics, Inc., equipped with a monochromatized X-ray source, installed at the Department of the Solid State Physics, Institute of Physics, University of Silesia. The samples were cleaned *in situ* in an ultra-high vacuum by etching with an Ar-ion beam with an energy of 2 keV. This enabled the removal of surface contamination originating from sample preparation and storage. The region of interest focused on isolated “blood vessels” about 200×800 μm in size.

## Results of Multiproxy Studies

### ”Vessels” Morphology and Microstructures

The “blood vessels” derived from the internal part of cortical bone from specimens WNoZ/s/7/166 and SUT-MG/F/Tvert/2 (Fig 1). The demineralized bone fragment revealed parallel-oriented “vessels” (fragments up to 2 mm long and about 50–100 μm wide) that bifurcate in some areas (Fig 1C–1G). They are preserved as reddish brown or yellow-orange, translucent, tube-like and branch-like floating structures (Fig 1A–1G). The vessels are mineralized with tiny crystals (1‒5μm, Fig 1I) of iron oxide, forming a coating that precisely mimics their external shape (Fig 1C–1H).

Thin-section analyses (SUT-MG/F/Tvert/2) based on optical microscopy, ESEM (Figs 1, 3A and 3C), and ToF-SIMS ions mapping (ToF-SIMS FIM; Fig 3D–3J) enabled us to make the following observations:

∎ The most intense ion signals from amino acid fragments of proline, hydroxyproline and leucine and others, Fig 3E–3I; were obtained from the vascular lumina of the blood vessels;
∎ The Fe^+^ ions and amino acids fragments overlap (compare distribution of ions on Fig 3D–3I);
∎ The most intense signals of iron derived from the outermost part of each of the studied “blood vessels”. Additionally, ion mapping indicated that iron ions seemed to be “scattered”, creating a form of an “agglutination” or “cloud” surrounding the vessel lumen (Fig 3D).

### X-Ray Diffraction

**Fig 4:**
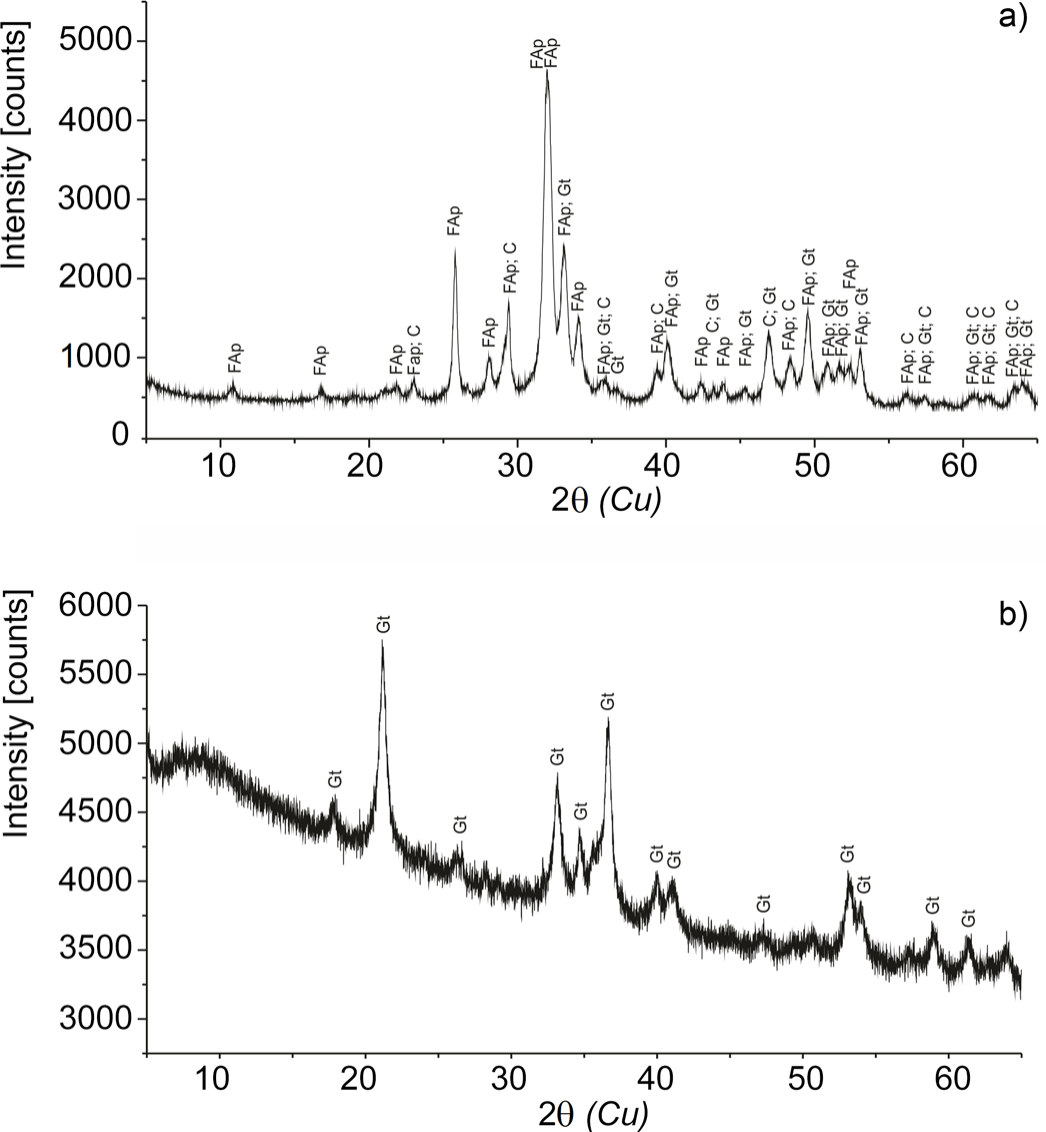
Powder X-ray diffraction of WNoZ/s/7/166 sample. a) Isolated blood vessels from sample WNoZ/s/7/166 and b) a fragment of cortical bone WNoZ/s/7/166. The bone consists mainly of fluoroapatite (95.4%), calcite (4.1%), and goethite (0.5%); however, in the case of isolated vessels, the crystalline phase of goethite occurred. FAp, fluoroapatite; C, calcite; Gt, goethite.

X-ray analyses revealed that powdered fossil bone samples (WNoZ/s/7/166, SUT-MG/F/Tvert/2) containing ferruginous layers are made mainly of fluoroapatite (95.3‒95.4%), and thus are typical diagenetically-altered fossil bones (Fig 4A). An admixture of goethite from 0.5 to 1.0 for both samples, as well as hematite (1%), was found in the SUT-MG/F/Tvert/2 sample. Isolated by EDTA incubation, powdered”blood vessels” from the WNoZ/s/7/166 sample (nothosaur humerus) showed the presence of only one crystalline phase: goethite (Fig 4B). XRD data from the recent control sample (marine iguana) indicated the presence of hydroxyapatite, a typical substance characterizing fresh, non-altered bones (S3 Fig).

### Infrared Analysis

**Fig 5:**
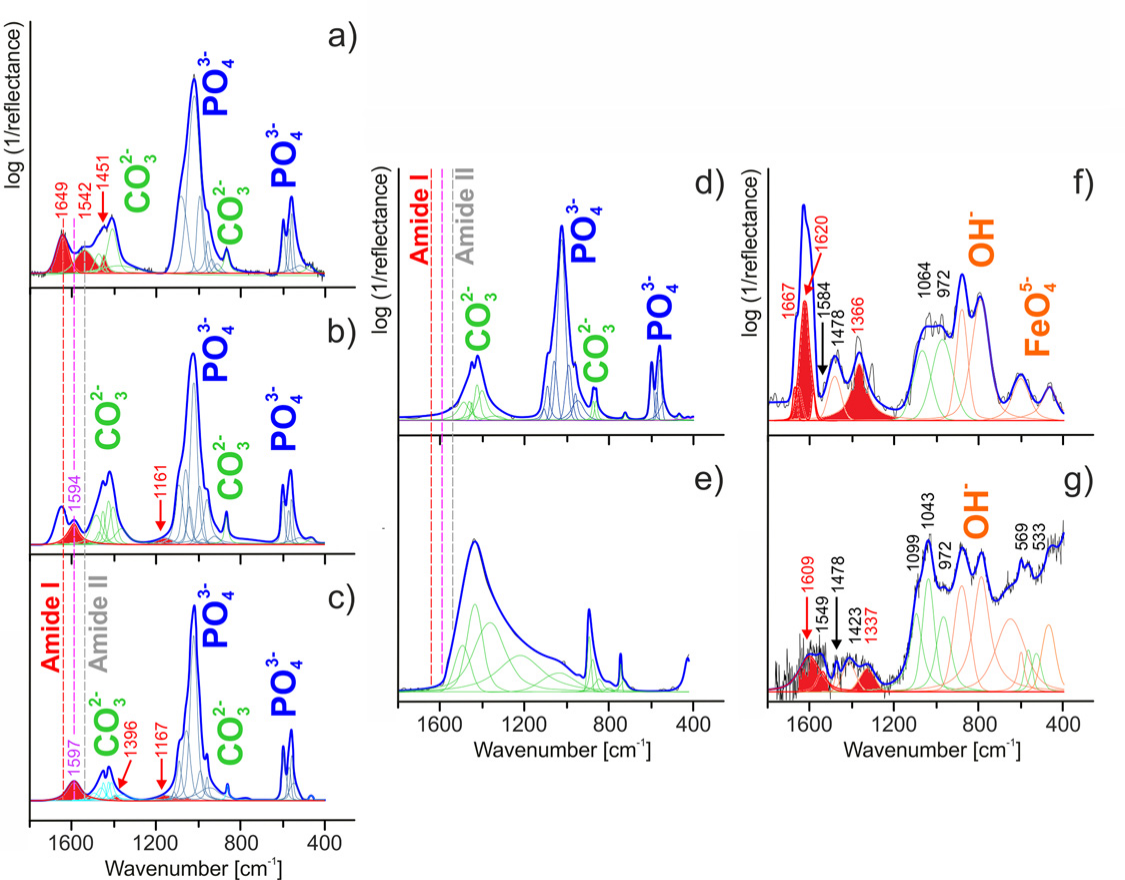
Infrared spectra of analyzed samples and control samples. Peak fit analysis based on the FTIR measurements for recent (a) and fossil bones (b‒d); pure carbonate (e); and two samples of fossilized blood vessels of WNoZ/s/7/166 (f and g). Each cortical bone (a‒d) shows an apatite (PO_4_)^3-^ as well as a carbonate (CO_3_)^2-^ peak, while both samples of WNoZ/s/7/166 infrared spectrum reveal a goethite (OH)^-^ and (FeO_4_)^5-^ peak. a) The primary cortical bone of a marine iguana femur shows amide bands at 1700‒1500 cm^-1^; b) and c) fossil bones of WNoZ/s/7/166 and SUT-MG/F/Tvert/2 reveal an amide peak at 1650‒1550 cm^-1^; d) the lack of amide components as detected in the nothosaur femur (SUT-MG/F/Tvert/15) bone; e) a sediment control sample from the vicinity of bone WNoZ/s/7/166; f) and g) isolated fossilized blood vessels show amide peaks at 1670‒1600 cm^-1^ and at 1450‒1200 cm^-1^. The other peaks come from amino acid residues and lipid structures.

The infrared spectra of fossil bone fragments (WNoZ/s/7/166, SUT-MG/F/Tvert/2, SUT-MG/F/Tvert/15) and recent marine iguana bone (GIUS-12-3628) are dominated by typical phosphate and carbonate attributed to fluorapatite (Fig 5A–5D) [42, 43]. In greater detail:

∎ the bands in the 1200‒950 cm^-1^ range are linked to anti-symmetric (ν_3_) and symmetric (ν_1_) stretching vibrations of (PO_4_)^3-^, whereas at lower wavenumbers, from 650 to 530 cm^-1^, out-of-plane (ν_2_) and in-plane (ν_4_) bending vibrations of phosphate units can be found.
∎ anti-symmetric (ν_3_) and symmetric (ν_1_) stretching vibrations of the carbonate (CO_3_)^2-^ ion are observed in the 1550‒1250 cm^-1^ range. Infrared bands in the 880‒750 cm^-1^ region originate from out-of-plane (ν_2_) and in-plane (ν_4_) bending vibrations of carbonate groups.

On the infrared spectra of “blood vessels” (WNoZ/s/7/166), bands in the region of 950‒750 cm^-1^ (OH modes) as well as below 650 cm^-1^ (vibration of ν(FeO)_4_^5-^) have been ascribed to the goethite phase (Fig 5F and 5G) [44, 45].

The other bands in the range from 1800 to 1100 cm^-1^ (fossilized bone material), as well as between 1650‒950 and 650‒550 cm^-1^, are assigned to organic material remains [42], [46, 47]. A more detailed analysis of organic regions (especially using the deconvolution process) enabled location and separation of the signals from amide, lipids, other organic remains, and phosphate and carbonate units. In general, the amide signal is associated with bands in the ranges of 3400–3030 cm^-1^, 1670–1200 and 650–550 cm^-1^ [48], while the presence of amino acid side chains and lipids are well marked over a wide wavenumber range from 1750 to 750 cm^-1^ [48–52]. The very weak signal/noise ratio in the case of bands ranging from 3500‒2800 cm^-1^ is difficult to interpret (see S4 Fig) and at the same time unambiguously due to overlapping of the hydroxyl group, molecular water and amide ones vibration (see S4 Fig). Hence, the infrared data imply that bands from the 3310‒3270 cm^-1^ region might be evidence of the NH stretching vibration coupled with the hydrogen bond of a carbonyl group in amide A, whereas infrared features ranging from 3100‒3030 cm^-1^ are associated with the NH stretching vibration of amide B, especially in case of sample WNoZ/s/7/166 (S4F and S4G Fig). Moreover, according to the literature, below 3000 cm^-1^ very weak bands might be related to the asymmetrical stretching mode of CH_3_ and CH_2_ groups in the case of lipids as well as NH_3_^+^ units linked to low molecular weight peptides or other organic residues [48]. Hence, our analysis was focused on data where signals from typical amide, amino acid and lipid regions are more clearly visible, i.e., below 1800 cm^-1^.

On the infrared spectrum of recent marine iguana samples (GIUS-12-3628), typical secondary amide structures were found at 1649 (amide I) and 1542 cm^-1^ (amide II) [18], [48], [53–56]. Amide I originates from the stretching vibration of C = O with minor contributions from CN, a CCN deformation and an NH bend [48], [54]. The amide II mode is related to the combination of the NH bend and the stretching vibration of CN with contributions from CO, CC and NC [48], [55] (Fig 5A). It is well known that all amide frequencies are conformation-sensitive, but amide I is the most widely used to determine protein conformations. Hence, we may attribute the α-helix secondary amide structure to the band at 1645 cm^-1^ [48]. Additionally, according to the band-fitting procedure, the band located at 1451 cm^-1^ can be ascribed to the CN stretching mode in proline [19], [48], [57] (compare Table 1). The bands corresponding to proteinaceous and lipid materials in Triassic fossil bones and blood vessels are summarized in Table 1. One important point worthy of consideration in conducting organic analysis based on infrared spectroscopy is the possible impact of bacterial and organic contamination which can be preserved in carbonate. For this reason, infrared analysis of bone material due to the strong vibration of ν_*asymm*_(CO_3_)^2-^ in the range of 1550‒1250 cm^-1^ is not completely unambiguous (Fig 5A–5C). To reduce the possibility of error during data analysis, SUT-MG/F/Tvert/15 without ferruginous coating (Fig 5D) was analyzed, as well as a sediment control sample (Fig 5E). In these cases, due to the high degree of degradation of the peptide, the amide signal was not observed. However, bands observed on the infrared spectrum of other Triassic bone samples (WNoZ/s/7/166, SUT-MG/F/Tvert/2; Fig 5B and 5C) at about 1594 and 1597 cm^-1^ originate from amino acid side chains [9], [17], [44]. Additionally, according to the literature, this band may correspond to the CC stretching vibration in tyrosine [44], [54], [58, 59] or to the bending amine mode in glycine [50], [59, 60]. In turn, bands located at 1396, 1167 (SUT-MG/F/Tvert/2), and 1161 cm^-1^ (WNoZ/s/7/166) are associated with, respectively, the bending of N^+^(CH_3_)_3_ [49, 50] or stretching of (COO^−^) in asparagine [48], [50], [59, 60] and the anti-symmetric stretching of CO-O-C in asparagine [48], [51, 52]. Amide components were also detected in the blood vessel sample (Fig 5F and 5G) mineralized with goethite. The blood vessel FTIR spectra justified by peak fit analysis in the 1670‒1200 cm^-1^ region demonstrate features characteristic of structural proteins. Typical amide bands were found at approximately 1667, 1620 (amide I), 1584 (amide II) and 1366 cm^-1^ (amide III) [61]. We infer that amide bands are associated with the stretching and bending vibrations of peptide (CO–NH) bonds. Moreover, due to protein conformations, the band at 1667 cm^-1^ may originate from turns secondary amide structure, while the band at 1620 cm^-1^ can be assigned to aggregated strands [48], [61]. Importantly, the three additional bands at 1478, 1064 and 972 cm^-1^ resulted in the presence of amino acid residues and lipid absorptions [48], [51, 52]. According to the data in the literature, the amino acids and lipids preserved in the”blood vessel” are ascribed to vibrations of:

- CH_2_/CH_3_ with an anti-symmetric character [48], [61] (1478 cm^-1^);
- NC/CC in trypsin [49] or C-O-P-O-C modes [61] (1064 cm^-1^);
- N^+^(CH_3_)_3_ with anti-symmetric stretching [61] (972 cm^-1^).

**Table 1:** Band assignments of organic signals for different samples (range up to 1800 cm^-1^).

Type of sample	Band location [cm^-1^]	Assignments
**GIUS-12-3628 recent marine iguan (powdered bone fragment, control sample)**	1649	Amide I (α-helix) ν(C = O)/ν(CN)/δ(CCN)/β(NH)
	1542	Amide II ν(NH)/ν(CN)/β(CO)/ν(CC)/ν(NC)
	1451	Amino acid side chains ν(CN) (pro)
**WNoZ/s/7/166 nothosaur humerus(powdered bone fragment)**	1594	Amide II, amino acid side chains ν(NH), ν(CC) (tyr)/ring (his)
	1161	Amino acid side chains ν(COO)^-^ (asp)
	1597	Amino acid side chains ν(CC) (tyr)
**SUT-MG/F/Tvert/2 tanystroph vertebra (powdered bone fragment)**	1396	Amino acid side chains δ_s_(CH_3_)
	1167	Amino acid side chains ν_as_(CO‒O‒C) (asp)
**SUT-MG/F/Tvert/15 nothosaur femur (powdered bone fragment, control sample)**	no data	not applicable
**WNoZ/s/7/166 extracted “blood vessels”**	1667	Amide I (Turns)
	1620	Amide I (Aggregated strands)
	1609	Amide I (Aggregated strands) (CO–NH)
	1584	Amide II
	1549	Amino acid side chains δ_s_(NH_3_^+^) (lys)
	1478	Lipid *δ*_as_(CH_2_)/*δ*_as_(CH_3_) *or* amino acid side chains ν(CN) (pro), δ(CH_2_), δ_as_(CH_3_)
	1423	Lipid δ_s_(CH_3_)
	1366; 1337	Amide III
	1099	Amino acid side chains ν(NC), γ(CH_2_)
	1064	Amino acid side chains and lipid ν(NC)/δ(CH)/ν(CC) (trp), ν(C‒O‒P‒O‒C)
	1043	Amino acid side chains ν(NC), γ(CH_2_)
	972	Lipid ν_as_(N^+^(CH_3_)_3_)
	533; 569	Lipid and amino acid side chains γ(CH_2_)

The organic signal observed on the infrared spectrum of WNoZ/s/7/166 (Fig 5F and 5G) is interpreted here as related to collagen or its degradation products. Amide I, in the form of aggregated strands, and amide III were assigned to bands located at 1609 and 1337 cm^-1^, respectively [48], [61]. It is known that many amino acid side chains absorb in or near the amide I, amide II, and amide III regions of the infrared spectrum. Hence, the amino acid side chains on the infrared spectrum might be represented by the NH_3_^+^ vibration of lysine [59], [61] or the CN vibration of proline or histidine [61] while the lipid absorptions are ascribed to the bending vibration of CH_2_ or stretching vibration of N^+^(CH_3_)_3_ [61]. The impact of oxidative deamination which cleaves the N–C covalent bonds typical of proteins should be taken into account in the case of fossil material. Thus, many amino acid and lipid structures were assigned to 1549, 1478, 1423, 1099, 1043 and 972 cm^-1^ (see Table 1). Moreover, the very low-lying bands at 569 and 533 cm^-1^ were linked to the CH_2_ vibration of lipids.

### High-Resolution Mass Spectrometry (ToF-SIMS)

**Fig 6:**
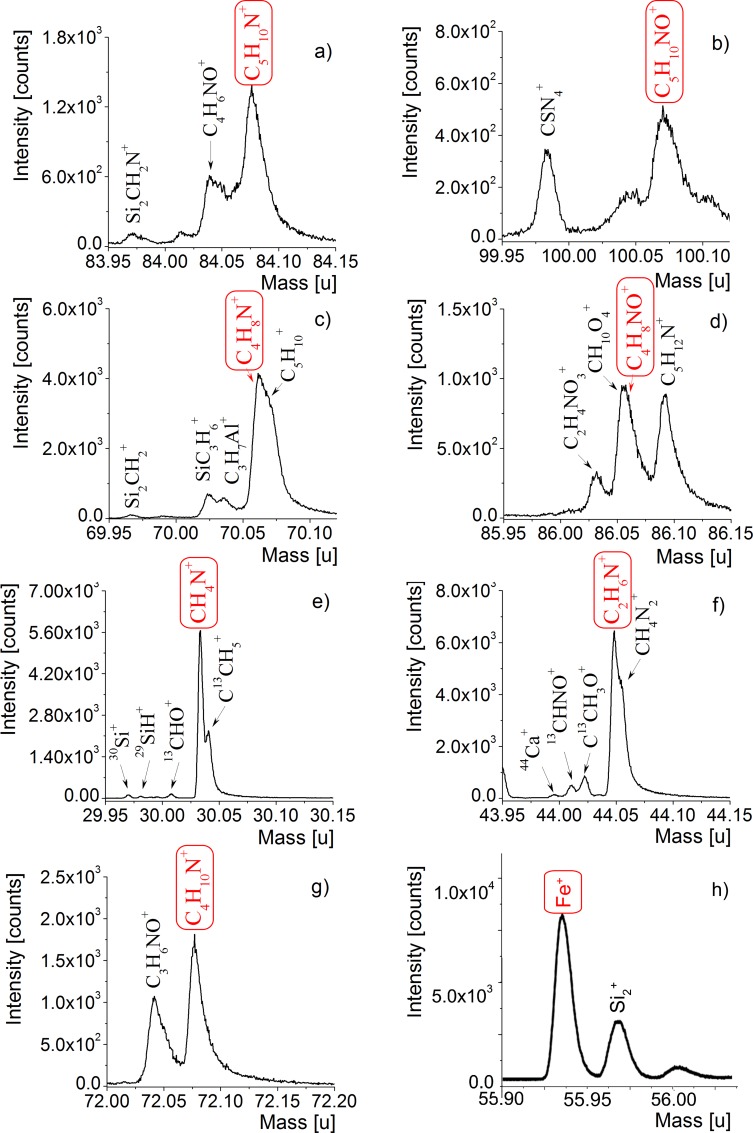
ToF-SIMS positive polarity spectra of fossilized blood vessel from demineralized WNoZ/s/7/166. The spectra show expanded *m/z* regions associated with amino acids. The chemical structures of the ions (in red, framed) corresponding to a) lysine, b) hydroxylysine, c) proline, d) hydroxyproline, e) glycine, f) alanine, g) leucine, and h) iron are shown in the spectra in panels (a‒h). Other nitrogen-containing organic fragments corresponding to amino acids are also shown.

Analyses of the ToF-SIMS spectra for both negative and positive polarity enabled the identification of a number of organic secondary ion species. The presence of various amino-acid-related nitrogen-containing species, for instance CH_2_N^+^ (*m/z* 28.02 Da), CH_3_N^+^ (*m/z* 29.03 Da), C_3_H_2_N^+^ (*m/z* 52.02 Da), NH_3_^+^ (*m/z* 17.02 Da), NH_4_^+^ (m/z 18.04 Da), CH_5_N^+^ (m/z 31.05 Da), C_2_H_2_N^+^ (*m/z* 40.02 Da), C_2_H_3_N^+^ (*m/z* 41.02 Da), C_2_H_6_N^+^ (*m/z* 44.05 Da), C_3_H_4_N^+^ (*m/z* 54.03 Da), C_3_H_8_N^+^ (*m/z* 58.06 Da), and C_3_H_4_NO^+^ (*m/z* 70.03 Da), observed in a mass spectrum is insufficient evidence to assign masses to specific amino acids. Similarly, in the case of research carried out for the detection of negative polarity arising from amino acids, specific ions, for instance NH^-^ (*m/z* 15.01 Da), NH_2_^-^ (*m/z* 16.02 Da), CN^-^ (*m/z* 26.00 Da), CH_2_N^-^ (*m/z* 28.02 Da), C_2_HN^-^ (*m/z* 39.01 Da), C_2_H_2_N^-^ (*m/z* 40.02 Da), CNO^-^ (*m/z* 42.00 Da), C_2_H_4_N^-^ (*m/z* 42.04 Da), CH_2_NO^-^ (*m/z* 44.02 Da), CHS^-^ (*m/z* 44.98 Da), CHO_2_^-^ (*m/z* 44.99 Da), C_3_H_3_O_2_^-^ (*m/z* 71.02 Da), C_3_HN_2_^-^ (*m/z* 65.02 Da), C_3_H_4_NO^-^ (*m/z* 70.03 Da), C_6_HN^-^ (*m/z* 87.01 Da), and C_6_H_3_N^-^ (*m/z* 89.03 Da), do not permit the clear identification of specific primary amino acids. Nevertheless, taking into account the presence of the several species CH_4_N^+^ C_2_H_6_N^+^ C_4_H_6_N^+^, C_4_H_8_N^+^, C_5_H_10_N^+^, C_4_H_8_NO^+^ and C_5_H_10_NO^+^, typically observed in amino acid mass spectra, we were enabled to conclude that organic compounds of glycine (CH_4_N^+^, *m/z* 30.03 Da), alanine (C_2_H_6_N^+^, *m/z* 44.05 Da), proline (C_4_H_6_N^+^, *m/z* 68.05 Da and C_4_H_8_N^+^, *m/z* 70.07 Da), leucine (C_4_H_10_N^+^, *m/z* 72.11 Da), and lysine (C_5_H_10_N^+^, *m/z* 84.08 Da), as well as hydroxyproline (C_4_H_8_NO^+^, *m/z* 86.06 Da) and hydroxylysine (C_5_H_10_NO^+^, *m/z* 100.08 Da) (Fig 6B and 6D) are present in the studied samples (compare in [9–11], [62–65]). Although CH_4_N^+^ (*m/z* 30.03 Da) is an ubiquitous amino-acid fragment, its presence next to fragments C_4_H_6_N^+^, C_4_H_8_N^+^ (attributed to proline), and C_4_H_8_NO^+^ (attributed to hydroxyproline) are very characteristic residues of type I collagen (compare [62], [66] and the literature cited therein).

We did not detect the presence of possible organic-associated ions in the sediment control sample from close proximity to bone (not shown in the paper). The ToF-SIMS spectra of bone matrix provided additional information about inorganic ions, for example residues of the bone apatite matrix, such as Ca^+^ (*m/z* 39.96 Da), CaO^+^ (*m/z* 55.96), Ca_2_PO_3_^+^ (*m/z* 158.89 Da), Ca_2_PO_4_^+^ (*m/z* 174.88 Da), and many others, including P‒O species (compare in [66]). Most organic ions are absent, including hydroxyproline and hydroxylysine fragments (compare S5 Fig). The very weak signal from the CH_4_N^+^ ion, along with that from the C_2_H_6_N^+^ fragment, may have originated in the intercellular spaces of bone matrix (compare S5 Fig).

### Photoelectron Spectroscopy

**Fig 7:**
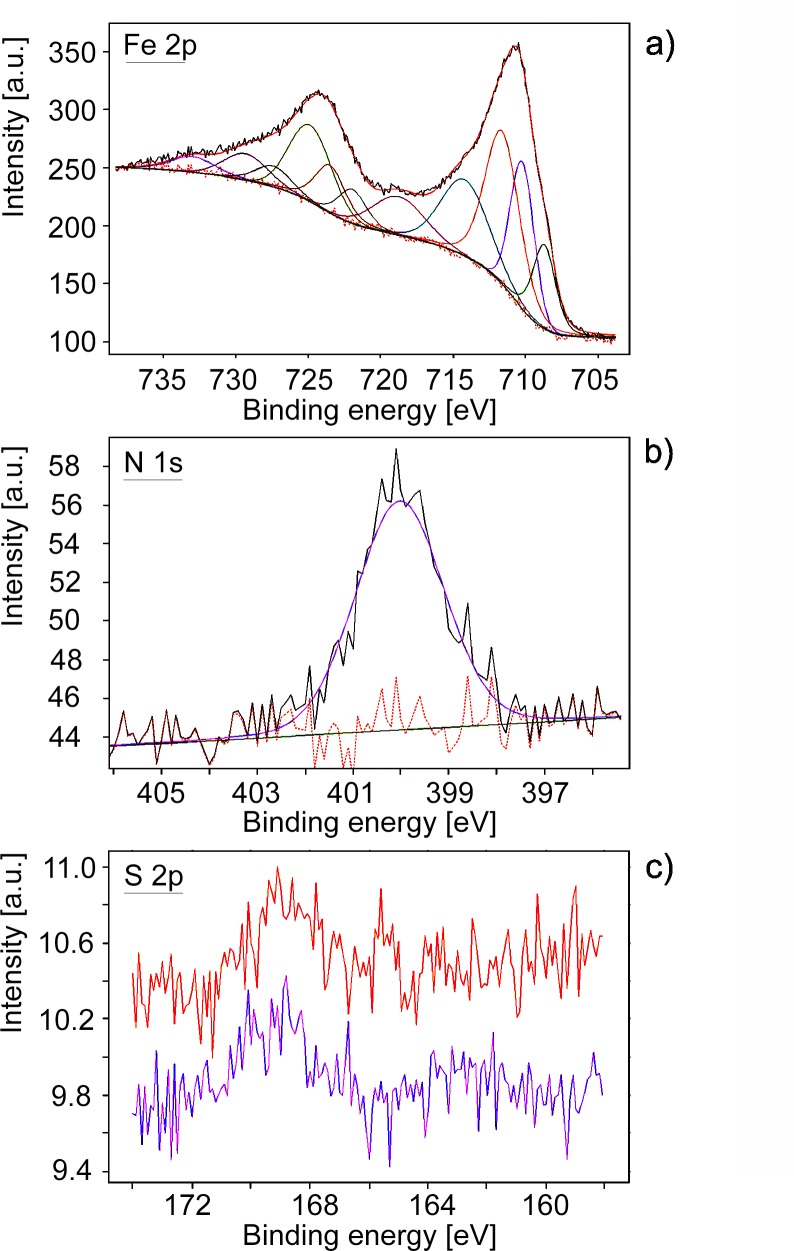
XPS survey data. a) Fe 2p multiplet obtained for isolated fossilized blood vessels together with the results of fitting; b) N 1s line obtained for isolated fossilized blood vessels together with the results of fitting; c) S 2p multiplet obtained for isolated fossilized blood vessels from two different spots in the same sample of blood vessel: red line: internal part of fractured fossilized blood vessel (corresponding to lumina); purple line: external part of fractured fossilized blood vessel (corresponding to vessel wall).

The XPS survey spectrum revealed oxygen, iron and carbon as the main components. Minor contributions to atomic concentration came from nitrogen, sodium, silicon, sulfur and phosphorus. In addition to their strong correlation with the results obtained from preliminary EDS investigations, the XPS measurements provided more complete information about the composition and chemical state of the analyzed surfaces. The atomic concentration of nitrogen with respect to iron is about 9%, while for sulfur it is 2%. The atomic O/Fe ratio is about 2.8. Analysis of the Fe 2p photoemission multiplet (Fig 7A) was performed in relation to the known experimental and theoretical data on Fe-O compounds [67]. The structure of the spectrum indicates the presence of trivalent iron ions and probably more than one component. The main components can be attributed to goethite and hematite [67]. The presence of a satellite peak with a binding energy of about 719 eV is characteristic for trivalent Fe compounds [67, 68], while the relatively strong component at 708.7 eV can be associated with divalent Fe ions, as in Fe_3_O_4_ [67, 68]. Taking into account the results obtained with other techniques, we can confirm that FeOOH is the main component containing iron, but other iron-containing compounds are present as well. This conclusion can be confirmed by the shape of the O 1s line, where more than two components characteristic for FeOOH were detected [69–71]. Analysis of the N 1s photoemission line shows the main component at 400.0 eV (Fig 7B). This binding energy is characteristic for amine or amide groups [72]. The structure of the S 2p multiplet indicates two chemical states of sulfur (Fig 7C), one with binding energy of about 163 eV, which is associated with organic matter, and another with a maximum at about 168.8 eV, characteristic of the oxidized state of sulfur [73]. In the control sample of recent marine iguana bone, the N 1s photoemission line and S 2p multiplet presented very similar values (not shown in the paper).

## Discussion

### Excluding Fungal and Microorganism Sources of Proteinaceous Organics

In 2008, Thomas G. Kaye and his coauthors (16) proposed that dinosaurian “soft tissues” found in fossil bones are bacterial biofilms which mimic real blood vessels and osteocytes. They suggested that microbial-mediated decay produced iron sulfides, which were later oxidized due to changing redox conditions. Therefore, in our study, special emphasis was placed on distinguishing mineralization of the original protein material of the bone from possible microbial contamination sources. Present-day studies on similar materials identified in bones of numerous taxa from the Cretaceous to the Recent [12], [19], [24], [74], as well as experimental approaches [26], support the endogeneity of the molecular material.

After an animal’s death, its bones are an attractive medium for diverse groups of microorganisms. Internal bone spaces such as medullary cavities and vascular channels can serve as migration paths for pore waters and mineral intrusions as well as biotic factors, for example fungi, hyphae and bacteria, which may introduce their own biomolecules (compare in [17]). Our examination of isolated fossilized “blood vessels” showed no microbial structures of any type in most analyzed samples. The absence of microbial activity traces, such as iron sulfides, especially pyritic framboids [75], as a product of microbial metabolism, and their oxidized forms, the pseudomorphoses [76] visible in optical or scanning electron microscopy, negate a significant impact by microbial contamination sources. Hyphae, filaments, and other spherical structures, both mineral and organic, are absent. There is no evidence of microborings on the outer bone surface or from fissures inside the bone.

The detection of hydroxyproline and hydroxylysine together in the analyzed material may indicate collagen as their potential source (compare in [9] and the literature cited therein). Although these amino acids may be included in proteins of various organisms, for the most part they occur separately. For instance, hydroxyprolines are found in plant glycoproteins [77, 78], and 2, 3-cis-3, 4-trans-3, 4-dihydroxy-L-proline, an analog of hydroxyproline, occurs in the cell walls of some diatoms [79, 80]. Hydroxylysine is also present in non-animal proteins, is a constituent of cell-wall-bound proteins in several groups of fungi [81], and can be formed by several bacteria [82], although the vast majority of bacteria cannot metabolize hydroxyproline (compare in [83]). Only some fungi and algae are able to produce both hydroxyproline and hydroxylysine [84]. However, our finding derives from dense, compact bone. Penetration of microbial and non-microbial (remnants of other organisms) contamination into the buried bone would have been severely limited, if not impossible. It is noteworthy that ToF-SIMS analyses of isolated fossilized "blood vessels" and vascular lumina on the thin section failed to detect fragments corresponding to biomarkers found in many microorganisms (compare in [85–87]).

### Preservation of Organic Molecules

We interpret the data presented here as evidence for the presence of organic residues in these specimens that may derive from collagen or its degradation products. Experimental approaches on thin sections of bone indicate that most of the bone collagen is removed by chemical rather than microbial degradation during the initial phase of bone diagenesis [88, 89]. Although protein degradation by bacterial activity in decaying bone can occur rapidly after death, does not progress rapidly, encompassing only 5 to 15% of the collagen present in fresh bone [89]. Moreover, bacterial removal of collagen takes place only from the outermost 20 to 30 μm of the bone [89], where organic matter is most exposed. The inner part of the compact bone is therefore protected from bacterial invasion to a much greater extent than its outer part.

X-ray diffraction and infrared spectroscopy showed typical chemical alterations of bone apatite during fossilization, expressed as a transition from hydroxyapatite to carbonate fluoroapatite (compare in [42]). However, this diagenetic alteration has not entirely degraded the primary organic matter originally forming the "blood vessels". The ToF-SIMS mass measurement and ion imaging, as well as XPS and FTIR data collected on the isolated and *in situ* (in thin section) “blood vessels”, indicate that organic residues are strictly limited to ferruginous coating of “blood vessels” and do not occur in bone apatite separately. Organic signals are present in the infrared spectra of powdered fossil bone fragments (compare Fig 5B and 5C); however, they are very weak, and overlapped by phosphate and carbonate peaks.

The significant amplification of organic signals in FTIR analyses appears after EDTA incubation, and thus after removing phosphate and carbonate phases from samples (compare Fig 5F and 5G), which confirms that they are strongly connected with ferruginous mineralization of “blood vessels”. The fixation of organic residues must take place during collagen gelatinization at a very early stage of diagenesis, since later physicochemical alteration of bone apatite seemed not to have much of an effect on organic preservation. It has been hypothesized that iron (hydro)oxides may enhance the preservation of organic molecules, thus preventing microbial or enzymatic degradation [12], [26]. Such a process of fossilization has been described from osteocytes in archaeological and fossil bones [74], and a chemical explanation for molecular and tissue “fixation” involving iron-catalyzed free-radical reactions without a role being played by bacterial decay was proposed by Schweitzer and her coauthors [26].

The marine iguana carcass was exposed to air after death, in contrast to the rapidly-buried Triassic bones. The remnants of partially removed or semi-dissolved collagen from the marine iguana, namely amide I (α-helix) at 1649 cm^-1^ and amide II at 1542 cm^-1^, are different from the iron-collagen cross links in fossil samples of “blood vessels”: amide I (turns) at 1667 cm^-1^, amide I (aggregated strands) at 1620 and 1609 cm^-1^, amide II at 1584 cm^-1^, and amide III at 1366 cm^-1^ and 1337 cm^-1^, respectively. Moreover, FTIR as well as ToF-SIMS studies of fossilized “blood vessels” indicate various amino acids functional for bone metabolism or formation of bone proteins and cell fluids, such as proline (CN stretching mode at 1478 cm^-1^ and C_4_H_8_N^+^, *m/z* 70.07 Da), glycine (bending amine mode at 1597 cm^-1^ and CH_4_N^+^, *m/z* 30.03 Da) as well as tyrosine, histidine, and asparagine ([61] and literature cited therein, see also [62]). Lipids have been also detected.

The mechanism of post-mortem iron-protein cross-linking proposed by Schweitzer and her colleagues [26] was also demonstrated by laboratory approaches. Additionally, it was shown [26] that iron oxides such as goethite may play an important role in both preserving and masking proteins in fossil tissues. The intimate association between organic molecules and iron in the studied bone samples is confirmed by SEM and ToF-SIMS imaging (compare Fig 3). Finally, the X-ray photoemission spectroscopy analysis helped to link molecular fragments identified by ToF-SIMS to bonding environments by confirming the presence of amide/amine bindings of nitrogen (Fig 7B). The low binding energy state of sulfur can be ascribed to sulfur-containing amino acids or to disulfides [10]. The analysis of the Fe 2p the photoemission line (compare Fig 7A) showed no iron state which could be ascribed to iron sulfide. Thus, the low binding energy state of sulfur (Fig 7C) can be ascribed to sulfur-containing amino acids.

The blurring of microstructural features in the analyzed demineralized samples (Fig 1) as well as intensive signals from potential collagen-associated amino acids in the vascular lumen in thin sections (Fig 3E–3I) may result from the progressive gelatinization and/or partial dissolution of collagen, processes that take place at an early stage of bone diagenesis [84]. Hydrolytic cleavage leads to fragmentation of bone collagen and its gelatinization and finally to collagen dissolution. Prior to complete dissolution, amorphous gelatin was leached by iron, triggering cross-linking, which acted as a fixative to stabilize the vessels. The source of iron for vessel mineralization could be heme and non-heme proteins, such as ferritin, derived from living cells and tissues (compare in [26], see also [90, 91]), since there were no sources of iron at all in the matrix surrounding the vessels. The breakdown of iron-bearing protein bonds releases iron, making it available for mineralization, and secondarily, remnant Fe^3+^ nanoparticles precipitate on the vessel walls [26]. This seems to be consistent with ToF-SIMS imaging, where the most intense iron signals form an “agglutination” or “cloud” around the vascular channels (compare Fig 3D), while “organic” signals occupied the vascular lumen. This phenomenon of rapid fossilization must have occurred during early diagenesis, most likely immediately after the death of the organism. Because the total body iron content of various animals varies over a range of 25–75 mg/kg [92], which may be insufficient for the precipitation of iron (hydro)oxides, external sources of iron cannot be excluded [93]. These iron-rich mineral casts are able to effectively protect fossil molecules over the long term.

## Conclusions

Our study provides clear evidence that fossil molecules could survive through rapid, early diagenetic iron radical cross-linking. These biomolecules could effectively be preserved in iron-rich minerals when the minerals precipitated directly onto soft tissues, such as vessels and cells, and tightly covered their original structure. It can be assumed that the persistence of protein remains of endogenous origin in Early Triassic bones was the result of early post- mortem mineralization processes on the walls of blood vessels. Our observations confirm the hypothesis, that iron oxides can act as protective envelopes enabling the preservation of endogenous biomolecules in dinosaur bones from the distant geological past [26]. This finding demonstrates that the possibility of the preservation of original soft tissue in iron-oxide mineral coatings may be greater than commonly believed and that molecules preserved in this way are structurally relatively undamaged and identifiable via spectral methods.

## Supporting Information

S1 Fig Geographical location. Geographical location. of outcrops and generalized geological section of Röt, Muschelkalk and Keuper in the Upper Silesia area. a) Geological section [after (*32*), strongly modified]; b) Map of Poland with location of outcrops.
(TIF)

S2 Fig Infrared spectrum of pure disodium ethylenediaminetetraacetate (EDTA). In the course of the analysis of the “blood vessel” samples, due to the demineralization process by EDTA, the EDTA spectrum was subtracted from the infrared spectra of vessels to completely eliminate the influence of this agent from the spectra and reveal the real component, i.e. part of the analyzed sample.
(TIF)

S3 Fig Powder X-ray diffraction of a recent marine iguana (GIUS-12-3628). The sample indicates poorly crystalline apatite corresponding to pattern #01-089-7834 (hydroxylapatite) with crystallites about 12 nm in size.
(TIF)

S4 Fig Infrared spectra of analyzed samples and control samples in the hydroxylated region. Peak fit analysis is based on the FTIR measurements for recent (a) and fossil (b‒d) bones; pure carbonate (e); and two samples of fossilized blood vessels of WNoZ/s/7/166 (f and g). In most cases (a‒c, f, g) distinguishing between an -OH group and amides A and B is difficult. In the control sample of nothosaurid femur (d), free of fossilized “blood vessels,” the typical amide signal from the region below 1800 cm^-1^ was not observed (compare Manuscript Fig 5D). Therefore the wide hump cannot be associated with any other amide in the region presented here. The signal/noise in the hydroxylated region for pure carbonate sample (host rock) is on very low level indicating lack of molecular water (e).
(TIF)

S5 Fig ToF-SIMS positive polarity spectra of bone matrix (SUT-MG/F/Tvert/2 sample). a) the spectrum from the range 20‒120 *m/z* corresponding to the range of occurrence of typical collagen-associated amino acid fragments, along with the juxtaposition of seven expanded *m/z* regions associated with amino acids as presented in Manuscript Fig 6; b) and c) weak signals from regions about *m/z* 30 (corresponding to CH_4_N^+^, *m/z* 30.03 Da) and about *m/z* 44 (corresponding to C_2_H_6_N^+^, *m/z* 44.05 Da) may have originated from intercellular spaces of bone matrix; d‒h) other regions corresponding to fragments as presented in Manuscript Fig 6, in detail: d) *m/z* 70.07 Da (C_4_H_8_N^+^), e) *m/z* 72.11 Da (C_4_H_10_N^+^), f) *m/z* 84.08 Da (C_5_H_10_N^+^), g) *m/z* 86.06 Da (C_4_H_8_NO^+^), h) *m/z* 100.08 Da (C_5_H_10_NO^+^), which may correspond to proline, leucine, lysine, hydroxyproline, and hydroxylysine, respectively. Note the lack of signals from other amino acids; i) comparison of ToF-SIMS spectra from the range *m/z* 55.92‒55.98 Da of bone matrix (black) and vessel wall (red) indicates the contribution of two different ions.
(TIF)
