# Supplementary figures and images for: Spectroscopic Studies on Organic Matter from Triassic Reptile Bones, Upper Silesia, Poland
Source: PLoS One. 2016 Mar 15;11(3):e0151143. doi: 10.1371/journal.pone.0151143 (PMC4792425; doi:10.1371/journal.pone.0151143)

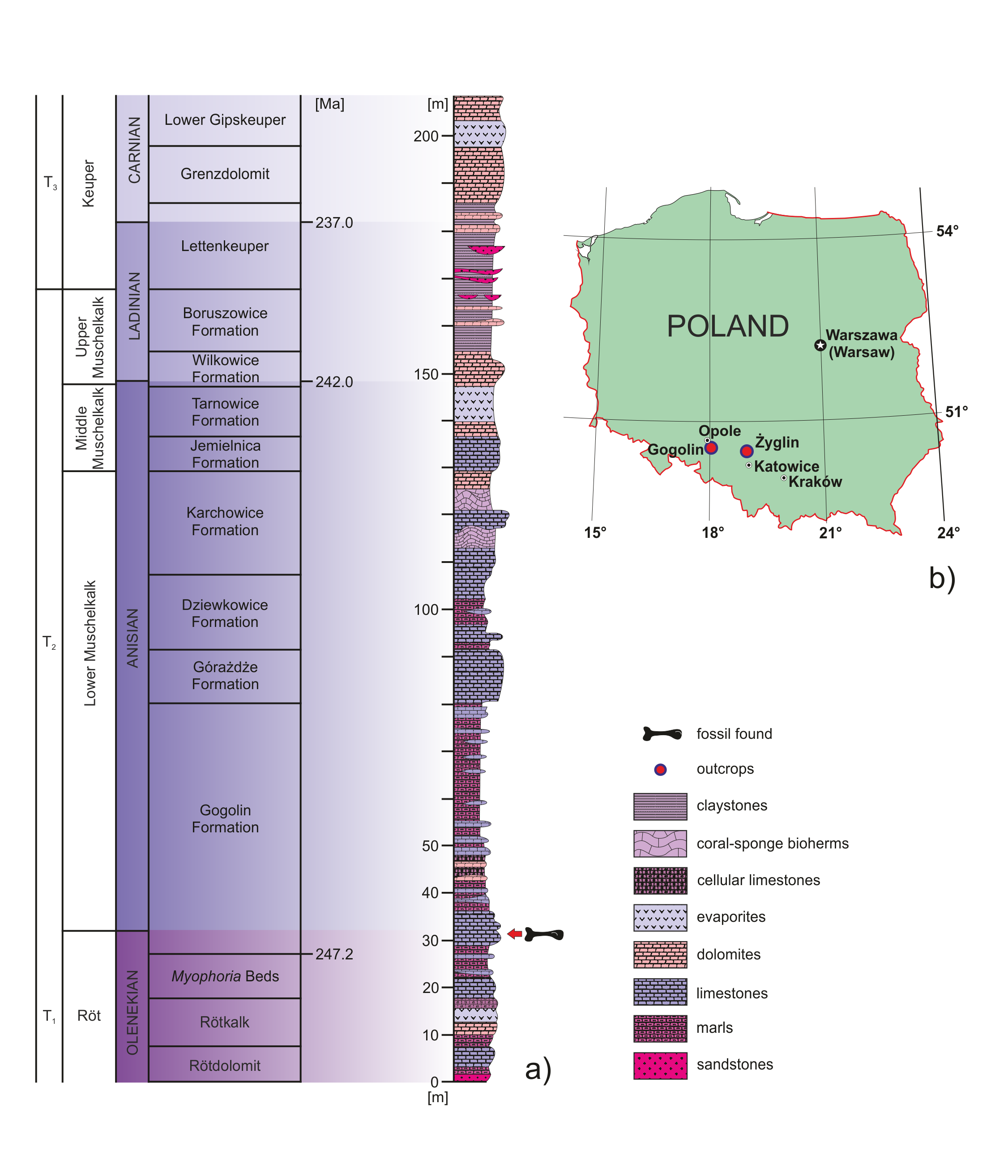

Supplement: S1 Fig — Geographical location. of outcrops and generalized geological section of Röt, Muschelkalk and Keuper in the Upper Silesia area. a) Geological section [after (32), strongly modified]; b) Map of Poland with location of outcrops. (TIF) [file pone.0151143.s001.tif]

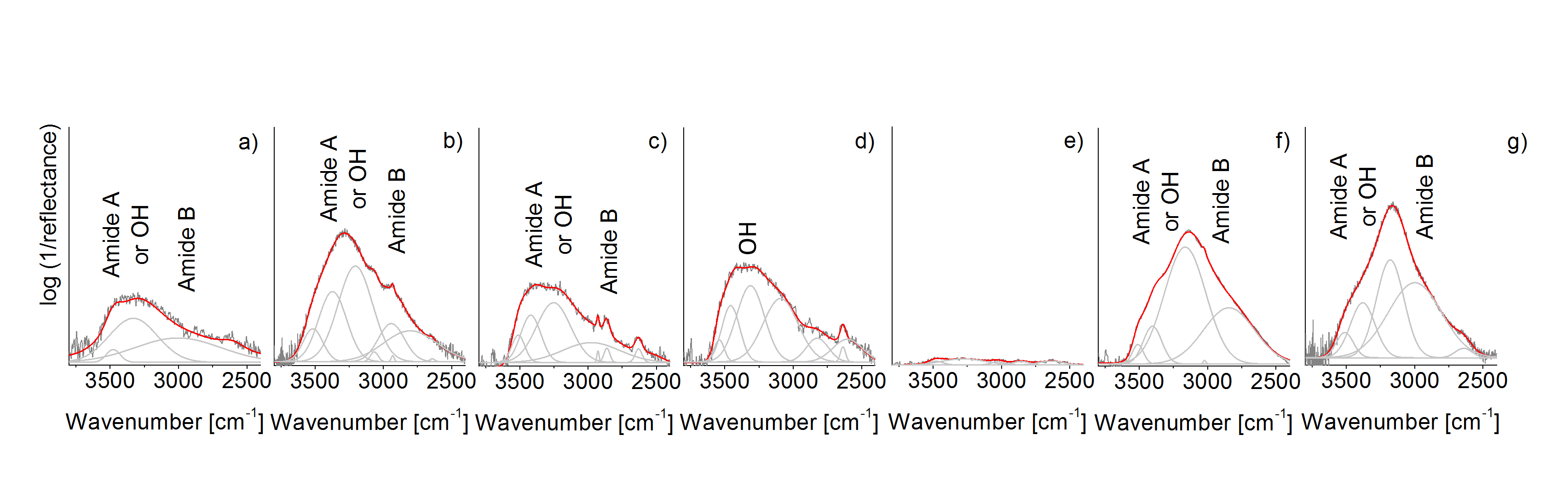

Supplement: S4 Fig — Peak fit analysis is based on the FTIR measurements for recent (a) and fossil (b‒d) bones; pure carbonate (e); and two samples of fossilized blood vessels of WNoZ/s/7/166 (f and g). In most cases (a‒c, f, g) distinguishing between an -OH group and amides A and B is difficult. In the control sample of nothosaurid femur (d), free of fossilized “blood vessels,” the typical amide signal from the region below 1800 cm-1 was not observed (compare Manuscript Fig 5D). Therefore the wide hump cannot be associated with any other amide in the region presented here. The signal/noise in the hydroxylated region for pure carbonate sample (host rock) is on very low level indicating lack of molecular water (e). (TIF) [file pone.0151143.s004.tif]

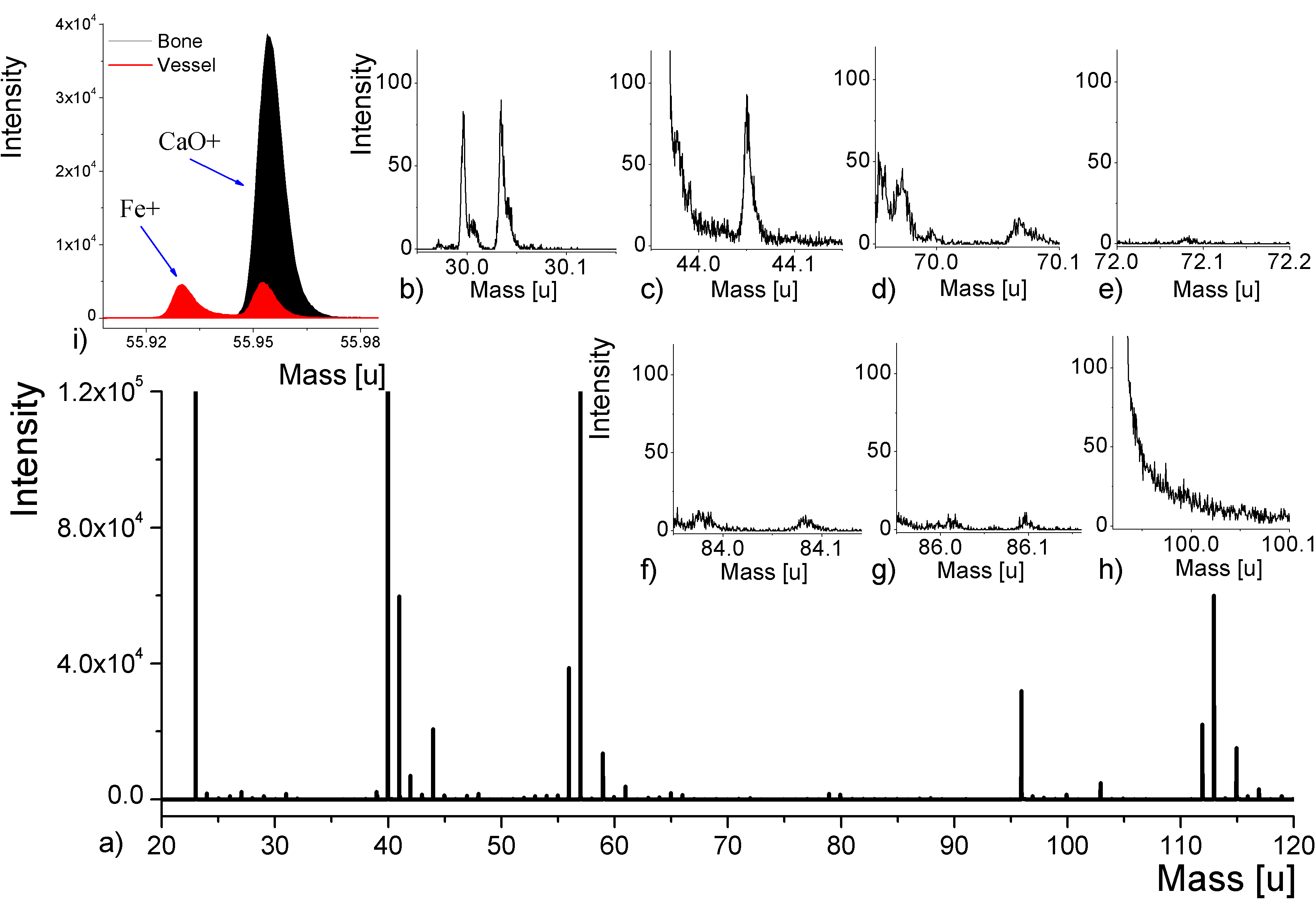

Supplement: S5 Fig — a) the spectrum from the range 20‒120 m/z corresponding to the range of occurrence of typical collagen-associated amino acid fragments, along with the juxtaposition of seven expanded m/z regions associated with amino acids as presented in Manuscript Fig 6; b) and c) weak signals from regions about m/z 30 (corresponding to CH4N+, m/z 30.03 Da) and about m/z 44 (corresponding to C2H6N+, m/z 44.05 Da) may have originated from intercellular spaces of bone matrix; d‒h) other regions corresponding to fragments as presented in Manuscript Fig 6, in detail: d) m/z 70.07 Da (C4H8N+), e) m/z 72.11 Da (C4H10N+), f) m/z 84.08 Da (C5H10N+), g) m/z 86.06 Da (C4H8NO+), h) m/z 100.08 Da (C5H10NO+), which may correspond to proline, leucine, lysine, hydroxyproline, and hydroxylysine, respectively. Note the lack of signals from other amino acids; i) comparison of ToF-SIMS spectra from the range m/z 55.92‒55.98 Da of bone matrix (black) and vessel wall (red) indicates the contribution of two different ions. (TIF) [file pone.0151143.s005.tif]
